# Purulent chondritis of thyroid cartilage

**DOI:** 10.1259/bjrcr.20200013

**Published:** 2020-06-02

**Authors:** Kim O Learned, Thinh H. Vu, Jeanie M. Choi, Maria K. Gule-Monroe, Kristen B. Pytynia, Lawrence E. Ginsberg

**Affiliations:** 1 Diagnostic Radiology, Neuroradiology (KOL, THV, MKG, LEG), and Head and Neck Surgery (KBP), University of Texas MD Anderson Cancer Center, Houston, TX, USA; 2 Diagnostic and Interventional Imaging, Neuroradiology, University of Texas Health Science Center, Houston, TX (JMC), USA; 3 Head & Neck Surgery, University of Texas MD Anderson Cancer Center, Houston, TX, USA

## Abstract

We report a case of a 77-year-old female with purulent chondritis of the thyroid cartilage who was initially referred for laryngeal neoplasm. Purulent chondritis of the laryngeal cartilage is a rare entity with three reports in the literature. The unique CT imaging features of expansile laryngeal cartilage with peripheral rim enhancement and central fluid-attenuation correlate to the abscess formation between the inner and outer perichondria. The correct imaging assessment prompts surgical management and avoid misdiagnosis.

## Clinical presentation

A 77-year-old female initially presented to her primary care physician with persistent dry cough and hoarseness and was diagnosed with post-nasal drip sinusitis. She was treated with oral antihistamine and a course of antibiotics. The patient did not improve and developed odynophagia and neck swelling, which prompted an otolaryngology referral. The otolaryngologist treated her for reflux and ordered a CT of the neck. The findings on CT of the neck were so alarming that the patient was directly admitted to a local hospital intensive care unit for airway monitoring overnight. She was discharged with antibiotics and steroids and referred to our cancer center with a presumptive diagnosis of laryngeal neoplasm.

## Investigations/Imaging findings

**Figure 1.**
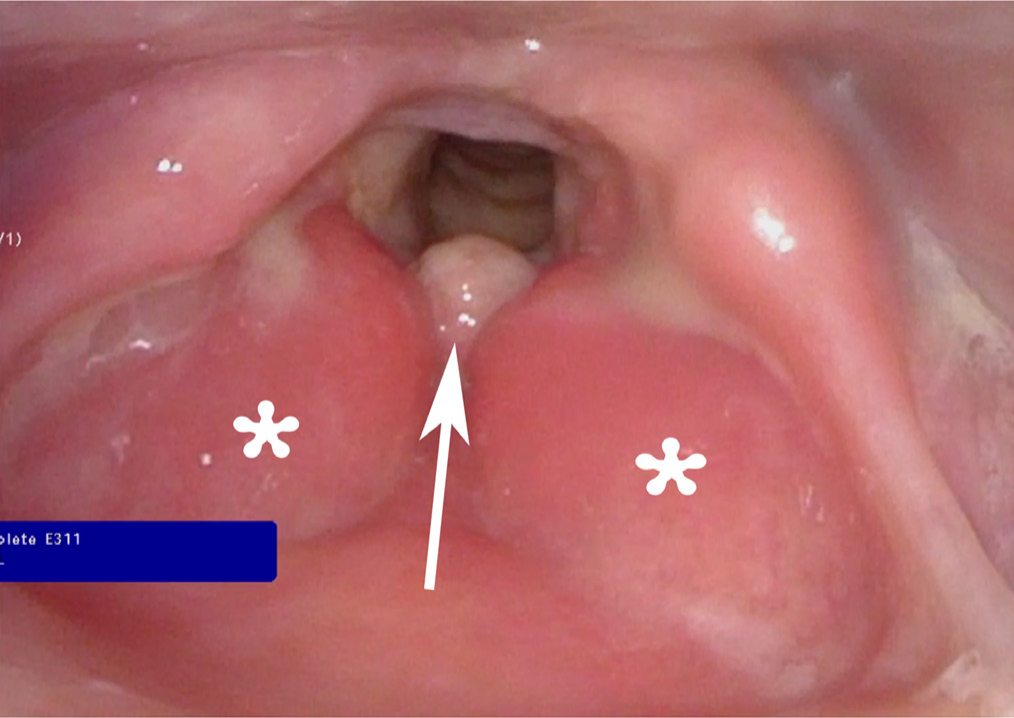
Photograph of laryngoscopy showed hyperemic edematous false vocal cords (asterisks) with a round lesion at midline petiole/anterior commissure (arrow).

At the initial consultation visit at MD Anderson Cancer Center, the patient reported 6 lbs. weight loss over the 6 weeks of hoarseness unresponsive to treatment. Her review of systems was negative for fever and night sweat. Her social history was notable for 10-pack-year tobacco smoking even though she quit 26 years ago. Her past medical history was unremarkable. Her physical exam revealed leukoplakia or oral thrush of the buccal mucosa, and flexible nasopharyngeal laryngoscopy was showed hyperemic and edematous vocal cords with a round lesion at midline petiole/anterior commissure (Figure 1).

**Figure 2.**
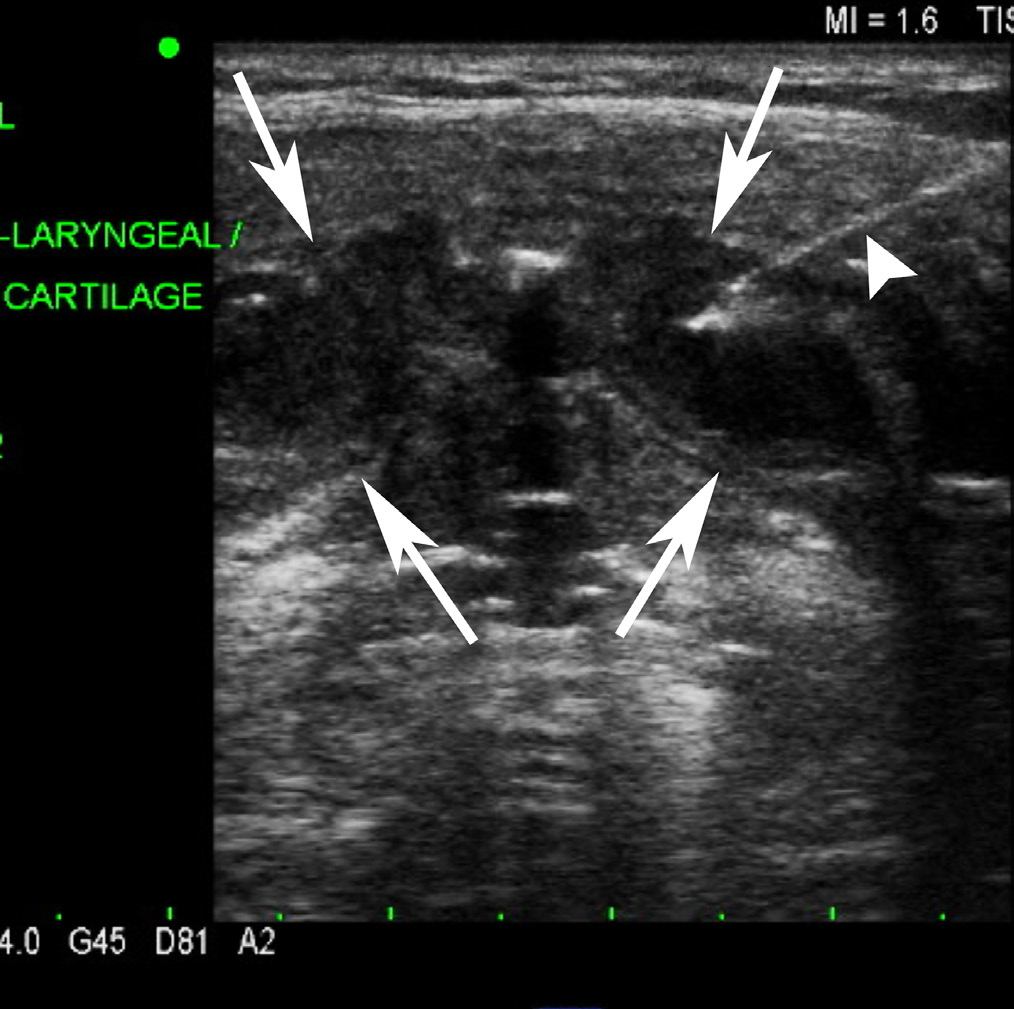
Transverse grayscale ultrasound image of the anterior midline neck at the level of larynx shows diffuse expansion of bilateral thyroid cartilage ala (arrows) with internal anechoic hypoechoic fluid and debris. An ultrasound-guided aspiration needle enters the left thyroid cartilage ala (arrowhead).

Our imaging work-up began with a neck ultrasound which identified diffuse expansion of the thyroid cartilage with internal anechoic hypoechoic fluid and debris (Figure 2). Ultrasound-guided fine needle aspiration (FNA) of the expansilethyroid cartilage yielded yellowish turbid aspirate. The FNA cytology showed abundant neutrophils and no malignant cells. Aspirate smear stains and cultures were negative for acid fast bacilli (AFB), microbacteria and positive for *Aspergillus* species, not fumigatus.

**Figure 3.**
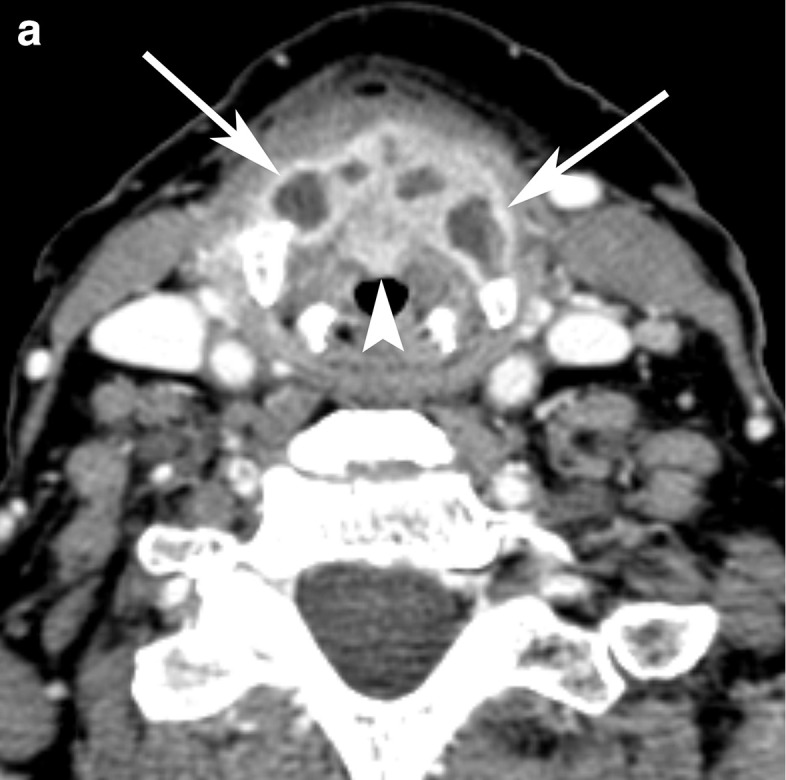
Pre-operative contrast-enhanced CT of the neck. (a) Axial and (b) coronal CT images through the supraglottis and thyroid cartilage show expansion of the thyroid cartilage (arrows) with internal hypoattenuation and peripheral rim enhancement, which is compatible with abscess formation between the perichondria. The anterior midline enhancing tissue (arrowhead) corresponds to the exophytic lesion evident on endoscopy at the petiole/anterior commissure.

A pre-operative CT of the neck was performed 1 week after the ultrasound and showed diffusely expanded thyroid cartilage with central fluid attenuation and peripheral rim enhancing tissue (Figure 3).

Blood tests included complete blood count, basic metabolic panel, *Cryptococcal* and *Aspergillus* antigen assay, *Coccidioides* antibody, rapid plasma reagin, human immunodeficiency virus type 1 and 2 RNA, T-spot Tuberculosis, C-reactive protein, sedimentation rate, and autoimmune panel and Histoplasma urine antigen test were unremarkable.

## Treatment

At direct laryngoscopy exam under anesthesia in the operating room, the 5 mm anterior commissure lesion was excised. An external incision and drainage of the anterior midline neck at the level of the thyroid cartilage was performed. The subcutaneous soft tissue and strap muscles were found to be extremely edematous and turbid material was released from thyroid cartilage and a small tissue biopsy was obtained. The anterior commissure pathology revealed granulation tissue, acute-chronic inflammation and necrosis, and the thyroid cartilage pathology demonstrated dense fibrous tissue with chronic inflammation. The specimen’s Gram stain and AFB stain were negative. Cultures revealed multiple oral flora and Scedosporium. An antifungal agent was added to the treatment regimen.

## Outcome and follow-up

**Figure 4.**
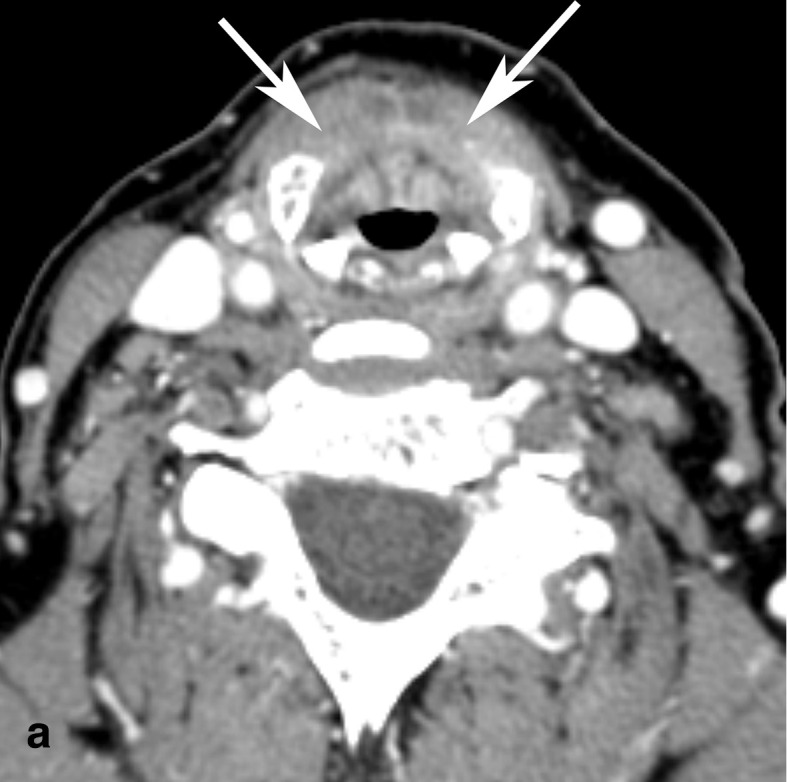
Follow-up contrast-enhanced CT of the neck. (a) Axial and (b) coronal CT images through the supraglottis and thyroid cartilage show normal ossification of the thyroid cartilage (arrows).

At 2-month follow-up, a CT of the neck demonstrated normal ossification of the thyroid cartilage with resolution of the prior lytic expansion and anterior commissure lesion (Figure 4). The patient’s hoarseness persisted but her voice had become stronger and laryngeal edema improved. Steroid treatment was discontinued and the patient completed another month of antibiotic and antifungal regimen without disease recurrence at 1 year follow-up.

## Discussion

Purulent chondritis of the laryngeal cartilage is defined as chondritis of the laryngeal framework cartilage with abscess formation between the inner and outer perichondria.^1,2^ The unique CT imaging features of laryngeal cartilage expansion withrim enhancement and central fluid-attenuation suggested an abscess between perichondria.^1,2^ The ultrasound also illustrated the anechoic hypoechoic fluid and debris of the abscess within the expanded thyroid cartilage, which was easily aspirated as yellowish turbid material on ultrasound-guided FNA. In this patient, the above-described unique imaging features with extensive thyroid cartilage involvement coupled with relatively mild symptoms of hoarseness and neck swelling that developed in a short time frame of 4–6 weeks was in keeping with the rare diagnosis of purulent chondritis of thyroid cartilage. The tubercular abscess is excluded by negative T-spot Tuberculosis test. The correct pre-operative diagnosis prompted appropriate surgical management with incision and drainage of the purulent thyroid cartilage.

Reported possible causes include prolonged intubation, relapsing polychondritisor other autoimmune disease, previous radiotherapy, trauma and idiopathic.^1,2^ Our patient’s positive cultures for *Aspergillus* species, not fumigatus and *Scedosporium* were considered normal flora and contamination as the patient did not have exposure risk factors, diabetes, prolonged steroid use or immune incompetence and all her serum tests were negative.^3,4^ As a result, the etiology remained idiopathic and it was difficult to discern whether we were dealing with a sterile specimen after several courses of antibiotics, an atypical infection, or a superimposed infection with an underlining malignancy in this patient with reported 10-pack-year smoking history.

The differential diagnosis for this rare entity includes invasive squamous cell carcinoma, chondrosarcoma, and rare autoimmune disease. The small size of the lesion in the anterior commissure appeared out of proportion to the large degree of extensively expanded necrotic destruction of the thyroid cartilage. In particular, the imaging features were unusual for advanced laryngeal carcinoma in patient without history of prior treatment.^5^

Laryngeal framework chondrosarcoma is less than 1% of all laryngeal tumors and affects males more than females in the sixth decade of life.^6^ The most common symptoms are hoarseness and dyspnea that can take up to an average of 28.1 months from onset to presentation. Laryngeal chondrosarcomas arise from the cricoid cartilage more commonly than the thyroid cartilage.^6^ Chondrosarcoma exhibits an expanded low attenuation appearance with stippled or coarse intra tumoral calcification.^5^ Our patient’s urgent 1–2 month onset and the diffuse thyroid cartilage involvement with central necrosis and peripheral rim enhancement without the ring-and-arc stippled chondroid matrix did not favor a chondrosarcoma diagnosis.

The common autoimmune diseases that can clinically present with laryngeal symptoms in adults include relapsing polychondritis and granulomatosis with polyangitis. Relapsing polychondritisis characterized byinflammation and destruction of different cartilaginous structures, including the ear, nose, larynx, trachea, bronchi, and peripheral joints and the heart’s connective components.^7^ The disease is associated with other autoimmune diseases in about 30% of cases.^7^ Laryngotracheobronchial involvement appears in nearly half of patients but the common clinical presentations are sudden onset of painful auricular and nasal chondritis, or polyarthritis. Typical CT findings of relapsing polychondritis include subglottic stenosis, tracheobronchial wall thickening with calcification with sparing of the posterior membranous wall, calcifications of the pinnae, and nasal cartilage collapse.^8,9^

Granulomatosis with polyangiitisis an idiopathic vasculitis of medium and small arteries, characterized by necrotizing granulomatous inflammation. The disease typically affects upper and lower respiratory tract with coexisting glomerulonephritis and is generally diagnosed by the presence of antineutrophil cytoplasm antibodies.^10^ Thesinonasal cavity is most commonly affected in the upper respiratory tract with mucosal thickening, soft tissue nodules and bone destruction. Therefore, our patient’s imaging features, clinical presentation and laboratory evaluation did not support an autoimmune etiology.

Note: Informed consent to publish could not be obtained from the patient and exhaustive attempts have been made to contact the guardian/next of kin of the deceased patient.

## Learning points

1. Recognizing the unique imaging features of purulent chondritisof the laryngeal cartilage is the key to the correct diagnosis, guiding prompt and appropriate clinical evaluation and management.
2. CT imaging depicts the unique imaging features of rim enhancing abscess in the cartilagewhich differ from theinvasive soft tissue squamous cell carcinoma,lytic expansilechondrosarcoma with ring-and-arc matrix or autoimmune entities.
3. Utrasound-guided FNA is useful for initial tissue biopsy and microbiology.
